# Effect of Stöber Nano-SiO_2_ Particles on the Hydration Properties of Calcined Coal Gangue-Blended Cement

**DOI:** 10.3390/ma17174218

**Published:** 2024-08-26

**Authors:** Nan Zhang, Hao Zhou, Yueyang Hu, Jiaqing Wang, Guihua Hou, Jian Ma, Ruiyu Jiang

**Affiliations:** 1 Key Laboratory for Advanced Technology in Environmental Protection of Jiangsu Province, Yancheng Institute of Technology, Yancheng 224051, China; zhangnanpaper@163.com (N.Z.); houguihua@ycit.cn (G.H.); 2 Nanjing Institute of Environmental Sciences, Ministry of Ecology and Environment of the People’s Republic of China, Nanjing 210042, China; zhouhao@nies.org; 3 Yancheng Institute of Technology, College of Materials Science and Engineering, Yancheng 224051, China; huyueyang1989@163.com; 4 College of Civil Engineering, Nanjing Forestry University, Nanjing 210037, China; jiaqingw@njfu.edu.cn

**Keywords:** cement, calcined coal gangue, Stöber nano-SiO_2_, hydration, compressive strength

## Abstract

This study focuses on the calcined coal gangue (CCG)-blended cements containing Stöber nano-SiO_2_ (SNS) particles. The effects of SNS particles on the workability, hydration behaviour, mechanical properties and microstructure evolution of the blended cements were comprehensively investigated at curing ages ranging from 1 to 28 d. The hydration behaviour was studied via isothermal calorimetry test, X-ray diffraction (XRD) and thermogravimetric (TG) tests. The microstructural evolution was studied using mercury intrusion porosimetry (MIP) and scanning electron microscopy (SEM). The results show that the incorporation of SNS led to a significant reduction in fluidity, particularly at an SNS content of 3%. The SNS significantly increased the compressive strength of the CCG-blended cement at all curing ages, and the optimum SNS content was found to be 2%. SNS significantly accelerated not only the early cement hydration but also the pozzolanic reaction of CCG at later curing ages, resulting in a decrease in portlandite, as evidenced by the isothermal calorimetry, XRD and TG analysis. Microstructural analysis shows that the incorporation of SNS effectively refined the pore structure of the CCG-blended cement, resulting in the formation of a dense microstructure. All these beneficial effects of SNS provides advantages in the development of the compressive strength of the CCG-blended cement at all curing ages.

## 1. Introduction

The production of Portland cement (PC) results in high consumption of natural resources and CO_2_ emissions. As a rough estimate, almost 0.930 tonnes of CO_2_ is produced for every tonne of cement clinker. The cement industry alone is responsible for about 8% of all the CO_2_ emissions worldwide, causing serious ecological and economic problems [1]. To achieve sustainable development in the cement industry, supplementary cementitious materials (SCMs) [2,3], including fly ash (FA), silica fume (SF) and granulated blast furnace slag (GBFS), are often blended to replace part of the cement clinker to reduce clinker factors, which is a common practice nowadays.

Coal gangue (CG) is a solid waste produced during coal mining and production [4]. The main mineral component of many CGs is kaolinite, which can be converted to metakaolin (MK) with high pozzolanic reactivity after high temperature activation [5]. This is commonly referred to as calcined coal gangue (CCG). As a potential pozzolanic material, CCG has been intensively studied for partial replacement of PC [6]. In this way, environmental problems such as the land occupation of CG and CO_2_ emissions from cement production can be alleviated to some extent [7].

The current literature reports that CCG can react with portlandite (CH) from cement hydration to produce additional calcium silicate hydrate (C-S-H) and calcium alumina silicate hydrate (C-A-S-H) gels [8,9]. These products are beneficial for refining pore structures [10], resulting in improved mechanical properties and durability of the mortar or concrete within an appropriate substitution range of 10–20% [5,11]. Yang et al. [10] investigated the compressive strength of mortars in which cement was replaced by different levels of CCG. Compared with the reference mortar, the 90-day compressive strength of the mortars prepared with 10% and 20% of CCG increased by 11.7% and 6.2% and the 180-day compressive strength by 16.9% and 11.4%, respectively. However, the corresponding early compressive strength was found to respectively decrease by 14.4% and 23.1% at 3 d and by 6.2% and 16.2% at 7 d. Furthermore, the authors [10] also claimed that further increases in the level of CCG substitution in cement would significantly weaken the strength development of the corresponding mortar. Similar observations were also reported in the work of Guo et al. [12], who studied the compressive strength of mortar prepared from 30 wt.% CCG with an optimal calcination condition of CG at 800 °C and a holding time of 2 h. They found that the compressive strength of the CCG-prepared mortar at 3, 7 and 28 d was 30.9%, 22.8% and 21.1% lower than that of the reference mortar, respectively. The current research indicates that although the inclusion of CCG up to 10–20% can ensure a satisfactory compressive strength at later ages, its strength development at early ages is much lower than that of the reference sample, which is very unfavourable for its popularization in engineering.

The cement paste is composed of calcium silicate hydrate (C-S-H) gel, portlandite, ettringite, numerous pores, etc., of which C-S-H accounts for about 70% of the cement paste [13]. The particle size of the C-S-H gel is about 10 nm, indicating that the hardened cement paste is a nanomaterial dominated by C-S-H. However, the microstructure of the hardened cement paste is very rough. In the past few years, nanomaterials have been applied in various scientific fields of engineering [14] and physics [15,16] to improve the micro and macro performance of materials. Due to their special ultrafine structures, nanomaterials exhibit many excellent properties such as grain small size effect, interfacial effect and quantum size effect [17]. Extensive research indicates that nano-SiO_2_ has been proven to effectively improve the performance of cement-based materials, especially in overcoming the problems of early slow performance development. In addition to providing nucleation sites for cement hydration, nano-SiO_2_ [18,19] can also react with Ca(OH)_2_ to form more C-S-H gel. The gel is conducive to the densification of the microstructure. Ultimately, the mechanical properties of cement-based materials are improved.

Of all the different forms of nano-SiO_2_ used to modify the properties of cement-based materials, Stöber nano-SiO_2_ (SNS) synthesized via the traditional Stöber method has a typical characterization of uniform and monodispersed spheres with high purity [20]. In the preparation process, controllable particle size and narrow size distribution can also be easily achieved [21]. Singh et al. [22,23,24] found that the addition of 5 wt.% SNS can not only increase the compressive strength of the control cement paste by 64% at 1 d and 35% at 28 d, but also reduce Ca leaching by 60% at 28 d. Oertel et al. [25,26,27] compared the effect of SNS and silica fume on the properties of ultra-high performance concrete (UHPC) and found that SNS can maintain its primary size with smallest agglomerates in UHPC. These monodispersed SNS particles were more conducive to achieving higher strength in UHPC. Similar findings were also found in the work of Sun et al. [28,29], where the calcined SNS can improve the compressive strength of PC and high-volume fly ash mortars better than the initial SNS. Although the relevant study of the effect of SNS on cement-based materials is limited in current research, it is still a promising method to keep nano-particles in a highly dispersed state and maximize the beneficial effect on the performance of cement-based materials.

Based on this, this study first investigates the effects of SNS content (from 0% to 3%) on the fluidity and strength development trends of Portland cement with 20 wt.% of CCG (PC-CCG). It then proceeds to discuss the potential mechanism of hydration in the SNS-added PC-CCG system using isothermal calorimetry, X-ray diffraction (XRD), thermogravimetric (TG), mercury intrusion porosimetry (MIP) and scanning electron microscopy (SEM). The knowledge gained from this research is expected to provide a new and in-depth understanding of the effect of SNS on the early age hydration performance of blended cement pastes containing CCG, thereby facilitating the practical application of CCG and promoting the sustainable development of the coal and cement industries.

## 2. Materials and Methods

### 2.1. Materials

**Figure 1:**
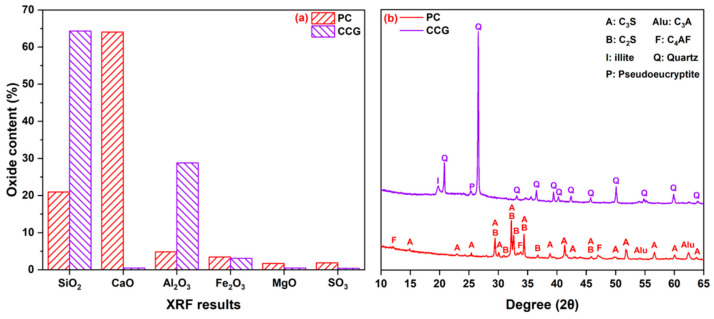
(**a**) Chemical composition and (**b**) mineralogical composition of raw materials.

**Figure 2:**
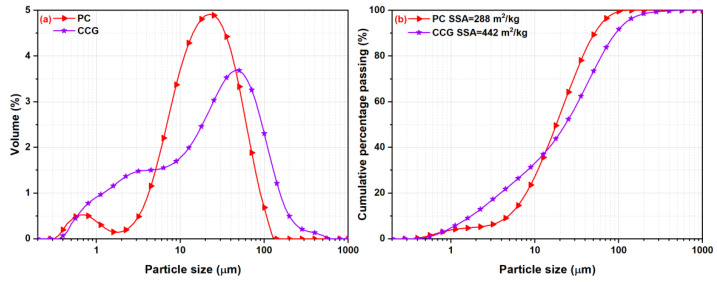
Particle size distributions of raw materials.

Portland cement (PC, Chinese P·I 42.5) and CCG were used for the preparation of blended cement pastes. CG sourced from Guizhou Province was subjected to drying, crushing, ball-milling and calcination processes to obtain CCG. The milling time was set at 15 min and the calcination was set at 800 °C for 1 h. Figure 1 displays the chemical and mineralogical compositions of PC and CCG. It can be seen from Figure 1b that tricalcium silicate (C_3_S), dicalcium silicate (C_2_S), tricalcium aluminate (C_3_A) and ferrite (F) are the main mineral phases in PC. Their particle size distributions are summarized in Figure 2. It can be seen that the D50 of CCG is smaller than that of PC, and their Blaine specific surface areas (SSAs) are 288 and 442 m^2^/kg, respectively.

**Figure 3:**
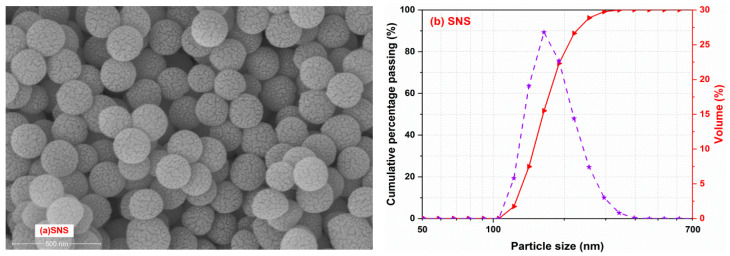
(**a**) Morphology and (**b**) particle size distributions of SNS.

Stöber nano-SiO_2_ particles (SNS) were first synthesized in the lab according to a classical Stöber method [20]. Deionized water, ammonium hydroxide and ethanol were mixed in a ratio using a magnetic stirrer followed by adding tetraethyl orthosilicate drop by drop. In particular, the experimental temperature and the mixing speed were set at 20 ± 1 °C and 800 rounds/minute, respectively. After synthesis, the above solution was centrifuged and the resulting solid calcined at 500 °C for 2 h [28]. Figure 3a shows the particle size and morphology of SNS. As observed, SNS particles show a uniform and monodisperse spherical shape and their average sizes are around 200 nm.

### 2.2. Samples Preparations

Table 1 shows the detailed mix proportions for the mortar samples in this experiment. As shown in Table 1, the blended cement consists of 80 wt.% PC and 20 wt.% CCG. In the process of using nan-SiO_2_ to modify cement-based materials, the amount of nan-SiO_2_ is usually controlled within 3% [18]. Therefore, in order to investigate the effect of SNS on the compressive strength of this blended cement, the replacement levels of SNS to PC in this experiment are set to 1 wt.%, 2 wt.%, and 3 wt.%, respectively. In addition, the water/solid (cement + CCG + SNS) ratio is kept at 0.5 throughout the experiment.

The raw materials weighed according to Table 1 were poured into the mortar mixing pot for low-speed mixing for 30 s, then the standard sand was added for low-speed mixing for 30 s, followed by high-speed mixing for a further 90 s. The prepared mortar was first tested for fluidity using the jumping table test, then poured into moulds of 40 mm × 40 mm × 160 mm and vibrated on the shaking table for about 1 min. Finally, the fresh mortar and moulds were placed in a standard curing room at 20 ± 1 °C for 24 h. After demoulding, the mortar specimens were immersed in water at 20 ± 1 °C until the compressive strength test at 1, 3, 7 and 28 d in accordance with GB/T17671-2021 “Test Method for Cement Mortar Strength” [30].

The preparation of paste samples was carried out to elucidate the underlying mechanism behind the variations in compressive strength of PC-CCG mortar resulting from the addition of SNS. The same mixture proportion to the mortar was used without the addition of sand. The weighed PC, CCG, SNS and water were mixed in a blender at 1600 rpm for 2 min, put into flat plastic tubes, gently shaken to shake out the potential bubbles introduced during the mixing process and covered with lids. Afterwards, the fresh paste and tubes were placed in a standard curing room at 20 ± 1 °C and ≥95% humidity until the microstructural analysis. At the time of testing, the cured paste was removed from the tubes, broken into small pieces and immersed in anhydrous ethanol to stop hydration (the anhydrous ethanol was updated once after 12 h). After 7 d of immersion, the piece samples were removed from the ethanol and dried in a vacuum drying oven at 40 °C for 1 d. Part of the small pieces was finely ground with an agate mortar until all powders could pass through an 80 μm square sieve for XRD and TG tests. Another part of the pieces was kept in the oven for MIP and SEM tests.

### 2.3. Methods

#### 2.3.1. Characterization of Mortar

The fluidity of the mortar was tested in accordance with GB/T 2419-2005 “Method for Determination of flow of cement mortar” [31]. The compressive strength was determined in accordance with GB/T17671-2021 “Test Method for Cement Mortar Strength” [30].

#### 2.3.2. Characterization of Paste

A TAM Air 8-channel microcalorimeter (Thermometric AB, Jarfalla, Sweden) was used to monitor the hydration heat behaviour of the hydrated paste. Approximately 4 g of fresh paste prepared in the paste sample preparation section was filled into the ampoule, sealed with a cap and placed in the calorimeter. Data were collected at a constant temperature of 20 °C for 3 d.

A MiniFlex 600 X-ray powder diffractometer (XRD, Rigaku, Tokyo, Japan) with a copper target (CuKα, λ = 0.154 nm) was used to determine the phase compositions of the hydrated paste. The working parameters were an accelerating voltage of 40 kV, accelerating current of 15 mA, scanning range of 5–65° and scanning speed of 5°/min. For qualitative analysis, the phases were identified with Search-Match using ICDD PDF-2.

A Mettler Toledo TGA/DSC1 synchronous thermal analyser (NETZSCH, Selb, Germany) with an accuracy of 0.1 mg was used to qualitatively and quantitatively determine the phase compositions of the hydrated paste by measuring the relative mass loss over a specified temperature range. The heating temperature range was 30–1000 °C, and the heating rate was 10 °C/min. The N_2_ atmosphere was used, and the inlet flow rate was 30 mL/min.

A PoreMaster 60 GT mercury injection apparatus (Quantachrome instruments, Boynton Beach, FL, USA) was used to evaluate the pore structures of the hydrated cement paste. The low pressure range was 0.2–50 psia and the high pressure range was 20–60,000 psia. Pore sizes ranging from 3 nm to 10.8 μm were able to be detected.

A Nova NanoSEM 450 scanning electron microscope (SEM, FEI Company, Hillsboro, OR, USA) was used to observe the morphological characteristics of the hydrated paste. Images were taken at an acceleration voltage of 15 kV, a working distance of 7–9 mm and a magnification of 2–20 k times. Prior to SEM analysis, the samples were coated with a layer of gold to improve their conductivity.

## 3. Results and Discussion

### 3.1. Fluidity

**Figure 4:**
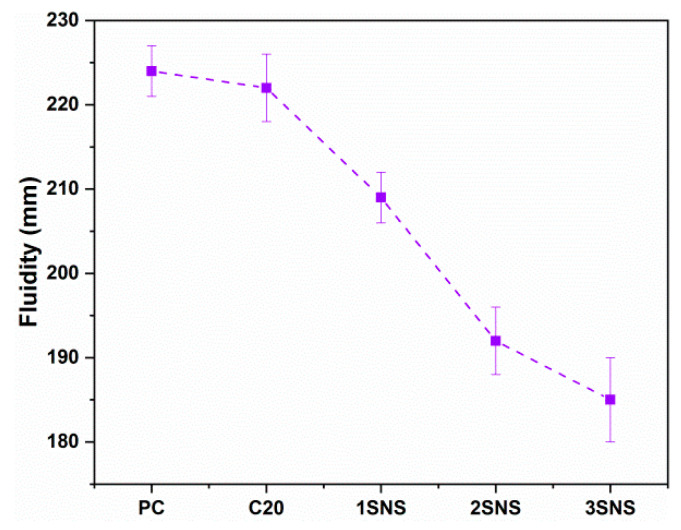
Fluidity results of all fresh mortars.

Figure 4 shows the fluidity of the fresh mortar samples. As shown in Figure 4, the fluidity of C20 was only slightly lower than that of the reference PC mortar, possibly due to the comparable particle size distributions of CCG and PC, which did not show much effect on the fluidity of PC, even though 20% of the cement was replaced by CCG. However, the fluidity of C20 was significantly reduced from 219 mm to 209 mm with the addition of 1% SNS. This is because the average particle sizes of the SNS particles are much smaller than those of the PC and CCG particles (Figure 2 and Figure 3), which contributes to the increased specific surface area and water needed to form lubricating layers around each particle. As a result, the C20 mortar with SNS experienced a reduced workability, particularly at 3% SNS, where the fluidity of C20 was reduced by 15.5%. It is also recognized by the current research that the introduction of nano-particles usually resulted in a sharp decrease in the workability of mortar or concrete [32,33].

### 3.2. Compressive Strength

**Figure 5:**
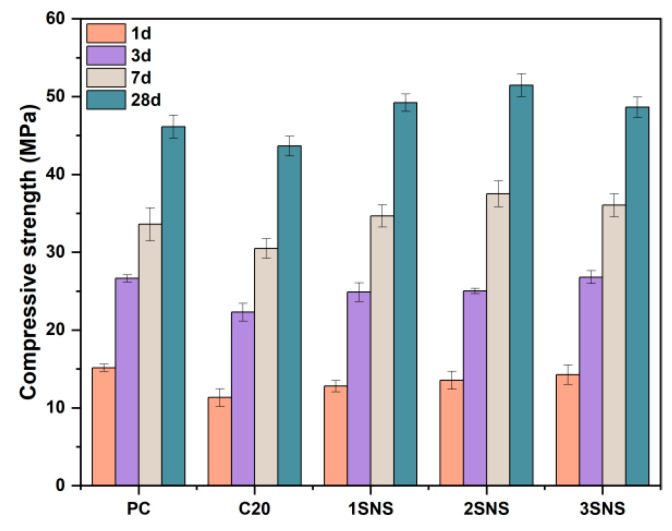
Compressive strength development of all mortars at 1, 3, 7 and 28 d.

Figure 5 shows the variation in compressive strength of all mortars developed from 1 to 28 d. As shown in Figure 5, the introduction of CCG decreased the compressive strength of PC mortar by 4.8 MPa after 1 d due to the much lower reactivity of CCG than that of PC. This is also consistent with other reports that when the CCG replacement level exceeded 10%, the strength development of PC-CCG mortar was severely inhibited within the first 1 d [34]. After 3 d, the compressive strength of PC-CCG mortar increased faster than that of PC mortar, and the difference in the strength values between PC-CCG and PC mortars was 4.3, 3.1 and 2.5 MPa at 3, 7 and 28 d, respectively. This can be explained by the starting reaction of CCG with CH from 3 d and the fact that more hydrates would be generated, especially at later ages. However, the increased quantity of hydrates in the PC-CCG system cannot fully compensate for the strength loss caused by the 20 wt.% reduction in the cement content.

When SNS was added, a clear trend was observed where increasing the SNS content significantly improved the compressive strength at 1 d. Compared to C20, the compressive strength of 1SNS, 2SNS and 3SNS improved by 12.9%, 19.4% and 25.9%, respectively. This is associated with the highly dispersed SNS particles, which can well exert the nucleation site effect that greatly accelerates cement hydration (as also will be discussed in Section 3.3), thereby effectively increasing the compressive strength of PC-CCG mortar [28]. Similar findings can also be observed at 3 d. However, it seems that the contribution of SNS gradually decreased, as reflected by the negligible strength differences between 2SNS and 3SNS mortars with the increase in the amount of SNS from 2% to 3% during this period. This observation is also consistent with the influence of other nanomaterials on the compressive strength of cement-based materials [35]. That is, the later the age of curing, the less the strengthening effect of the nanomaterials on the strength development.

From 7 d onwards, all the SNS-added mortars showed higher compressive strength than the reference mortar. As shown in Figure 5, the compressive strength of 1SNS, 2SNS and 3SNS mortars reached 34.6, 37.5 and 36.1 MPa after 7 d, which were 3.1%, 11.7% and 7.3% higher than those of the reference PC mortar, respectively. At 28 d, the corresponding increases were still as high as 6.7%, 11.5% and 5.4%, respectively. These values indicate that the addition of SNS can fully compensate for the loss of strength caused by the addition of 20 wt.% CCG from 7 to 28 d, the reason for which will be discussed in the following section. In addition, it is important to note that the SNS content should not exceed 2%, as this can lead to a stagnation of the strength growth in the medium-to-late stages of the mortar.

In order to better understand the mechanism of the introduction of SNS to improve the compressive strength of the PC-CCG system and to simplify the research scheme, the systems of PC, C20 and 2SNS were selected for further hydration study.

### 3.3. Isothermal Calorimetry

**Figure 6:**
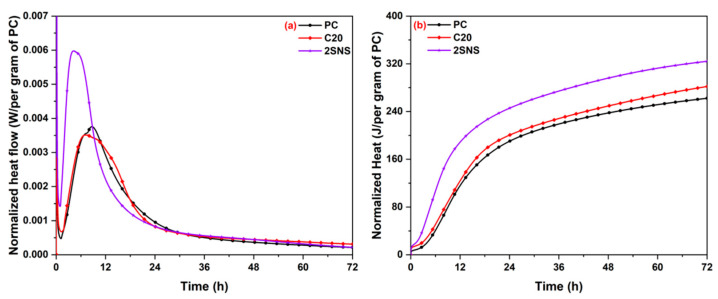
(**a**) Heat flow and (**b**) cumulative heat release of the selected pastes.

Figure 6 shows the hydration heat evolution of PC, C20 and 2SNS pastes, normalized per gram of cement. As shown in Figure 6a, the heat flow curve of PC paste had two exothermic peaks [36]. The first peak was mainly associated with hydration of alite and the second one was related to the renewed formation of ettringite (AFt), appearing at 7.2 h and 8.8 h, respectively. The addition of CCG exhibited a hydration heat curve with a trend comparable to that of PC during the acceleration stage, and the first peak was observed at about 6.9 h. The C20 sample, although containing only 80 wt.% cement, exhibited a heat release rate comparable to that of the PC sample, indicating that CCG greatly accelerated cement hydration. When 2 wt.% SNS was added, the corresponding paste showed a noticeably shorter induction period compared to the PC and C20 pastes, with the first peak appearing at 4.1 h. In addition, the slope of the C_3_S exothermic peak increased in the SNS-added samples, and this peak also occurred at an earlier stage and had a higher heat flow. All these observations suggest that the addition of SNS significantly enhances cement hydration.

Figure 6b presents the cumulative hydration heat curves of samples within 72 h. The total heat release of PC was 262 J/g. For the C20 and 2SNS samples, the total heat of samples experienced an increase of 7.6% and 23.7%, respectively. The results suggest that the presence of SNS can significantly enhance the hydration process of cement. This acceleration effect is attributed to a nucleation effect of SNS [28] caused by its significantly higher surface area than PC and CCG. In addition, the acceleration of cement hydration can result in the rapid formation of a dense microstructure, thereby improving the compressive strength of the cement. This is in good agreement with the strength data, as evidenced by the significant increase in the 1- and 3-day compressive strength data of the CCG-prepared mortar after the addition of SNS in Figure 5.

### 3.4. XRD Analysis

**Figure 7:**
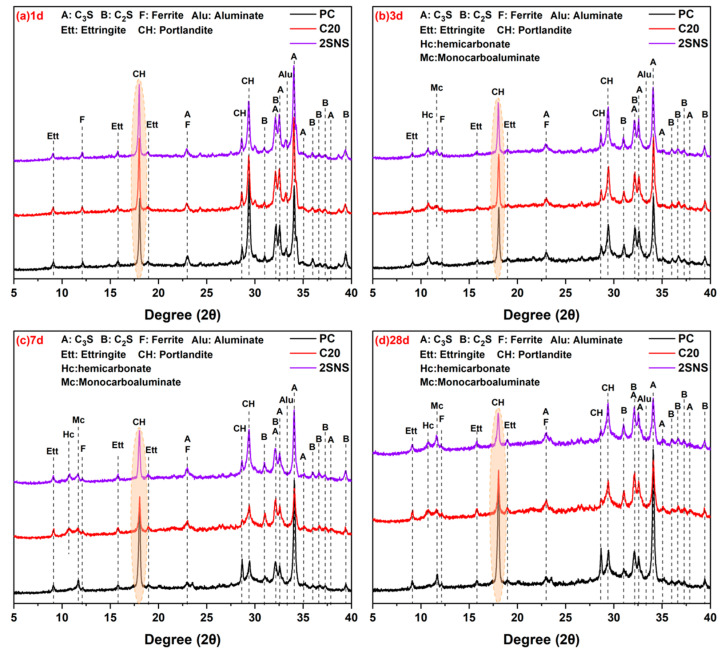
XRD patterns of the selected pastes at (**a**) 1 d, (**b**) 3 d, (**c**) 7 d and (**d**) 28 d.

Figure 7 shows the XRD patterns of hydrated cement pastes at different curing ages. The main hydration products of PC, as shown in Figure 7, are portlandite (CH) and ettringite. CH is formed by the hydration of the silicate phase in PC. It can be seen that its peak intensity (2θ 18°, oval area in Figure 7) showed an increasing trend with increasing curing age due to the continuous hydration of the silicate phase. Ettringite is formed by the hydration of the aluminium phase. After the incorporation of CCG, portlandite and ettringite remained as the main hydration products. This indicates that the presence of CCG did not alter the type of hydration products of PC. At 1 d, the characteristic peak intensity of CH in the C20 sample increased compared to that in PC, indicating that the addition of CCG promoted cement hydration, which is consistent with the results of the hydration heat observation in Figure 6a. After 3 d, there was a decrease in the intensity of the characteristic peak of CH in the C20 sample, which can be attributed to the pozzolanic reaction of CCG resulting in the consumption of portlandite, particularly after 7 d (Figure 7b). This reaction between CCG and CH produced additional C-A-S-H that can support the strength development of C20, aligning well with the analysis of a narrowing strength gap between C20 and PC (Figure 5).

In the presence of SNS, the primary hydration products consisted of portlandite and ettringite, which were identical to those observed in PC. The peak intensity of CH showed an increasing trend and the peak intensity of the mineral phase (2θ 18°) of the clinker showed a decreasing trend after the addition of SNS during early curing ages. This can be attributed to the accelerating effect of SNS, which is consistent with the hydration heat data. Compared with C20, the peak intensity of CH decreased significantly at later curing ages (especially at 28 d in Figure 7d), which can be attributed to the acceleration of the pozzolanic reaction by SNS.

### 3.5. TG Analysis

**Figure 8:**
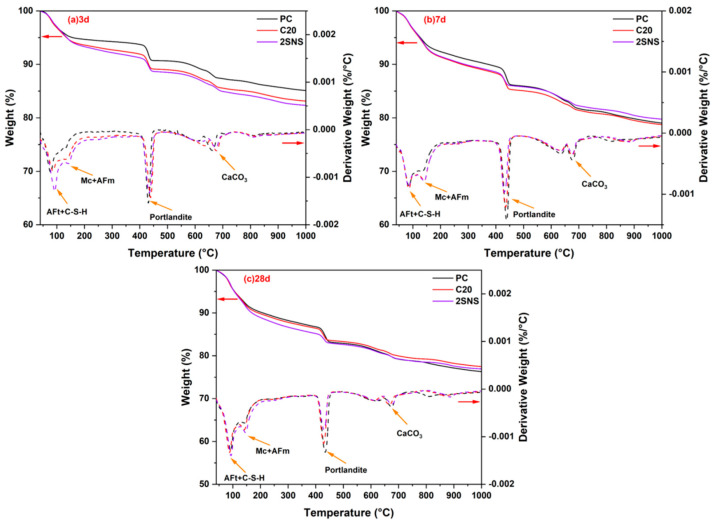
TG−DTG curves of selected pastes at (**a**) 3 d, (**b**) 7 d and (**c**) 28 d.

The TG−DTG results of the selected pastes after 3, 7 and 28 d of hydration are shown in Figure 8. From the DTG curves, it can be seen that four major mass loss peaks were observed during tests [37]. The first mass loss peak between 50 and 100 °C was mainly associated with the dehydration of calcium silicate hydrate (C−S−H) and ettringite. The second mass loss peak between 130 and 200 °C was related to the dehydration of carboaluminates (Hc and Mc) and AFm phase. The third peak between 400 and 500 °C was due to the decomposition of the CH. The mass loss in the range 600−800 °C was mainly due to the decarbonation of CaCO_3_. The DTG analysis showed that C-S-H, AFt, Mc, carboaluminates and CH were the common hydrates present in all samples. The crystalline hydrates observed from DTG curves were found to be in good agreement with the results obtained from XRD analysis (Figure 7).

The area of the weight loss peak in the DTG curve reflected the weight of the hydration products. From 3 d to 28 d, the weight loss peak area in the range 50−200 °C of the SNS sample was observed to be the largest among the selected pastes (Figure 8a), indicating that it generated the highest quantity of hydration products. This is due to the fact that the addition of SNS resulted in the acceleration of hydration. However, at 7 d and 28 d, the weight loss peak area in the range 400−500 °C of the SNS sample was observed to be the smallest among the selected pastes. At later curing ages, the pozzolanic reaction of CCG consumed CH, leading to a reduction in its weight loss. When SNS was added, the pozzolanic reaction of CCG was accelerated. Consequently, it can be observed that the selected pastes exhibit the smallest weight loss peak area for CH (Figure 8b,c). The observation is also in line with the XRD analysis (Figure 7c,d).

### 3.6. Pore Structure Analysis

**Figure 9:**
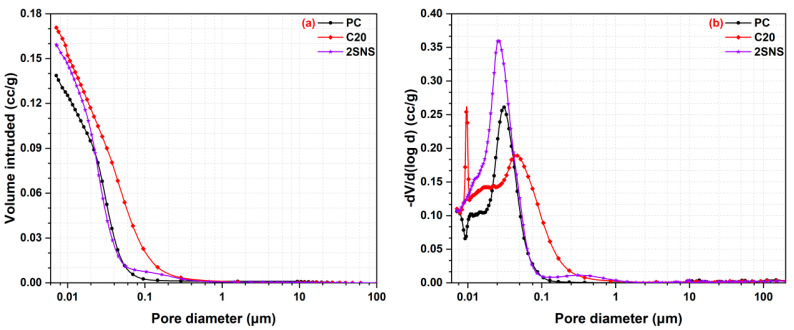
(**a**) Cumulative pore volume and (**b**) differential pore size distribution curves of the selected pastes at 28 d.

The pore structure, including porosity and pore size distribution, is crucial to support the performance of cement-based materials. Figure 9a shows the cumulative pore volume curves of PC, C20 and 2SNS pastes at 28 d. It can be seen from Figure 9a that both C20 and SNS had much higher porosities than PC at 28 d due to the significantly decreased gel-to-space ratio when 20 wt.% cement was replaced by CCG. In addition, the total porosity of C20 and SNS was 17.1% and 15.9%, respectively. This indicated that SNS effectively reduced the porosity of C20 at 28 d.

**Figure 10:**
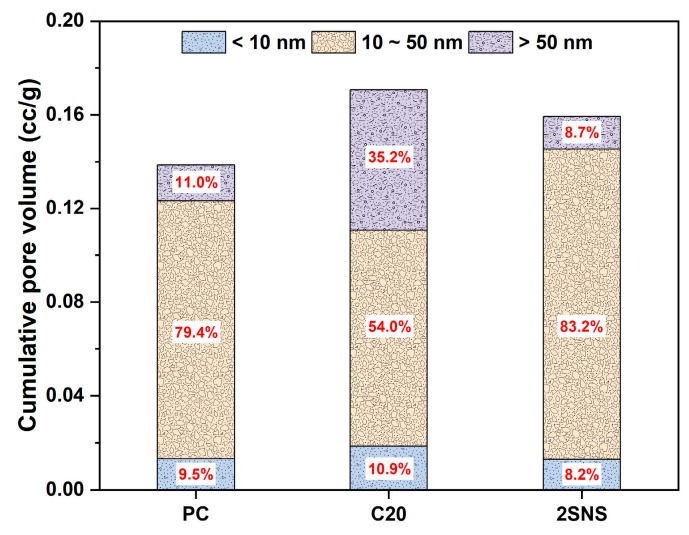
Volume fractions of different types of pores in the selected pastes.

As shown in Figure 9b, the pores in these pastes can be classified into three types: gel pores (<10 nm), medium capillary pores (10–50 nm), and large capillary pores (>50 nm). The medium capillary pores were beneficial to the strength and permeability of cement-based materials, while the large capillary pores could deteriorate the relevant properties [38]. According to the classification of these types of pores, the volume fractions of different types of pores were calculated and shown in Figure 10.

It can be seen that the volume fractions of large capillary pores in C20 and SNS-added paste were 35.2% and 8.7%, while the volume fractions of the medium capillary pores in these two pastes was 54.0% and 83.2% at 28 d, respectively. This can be explained by the promotion effect of SNS on the cement hydration and the CCG reaction that produced more hydrates than that in C20, which filled the large capillary pores. Through the analysis of the pore structure, it was found that the addition of SNS could refine the pore structure of C20, as evidenced by the decreased porosity and large capillary pores and the increased medium capillary pores. This refining effect of SNS on the pore structures of C20 also well explains the improved compressive strength development of C20 with the addition of SNS at 28 d.

### 3.7. SEM Analysis

**Figure 11:**
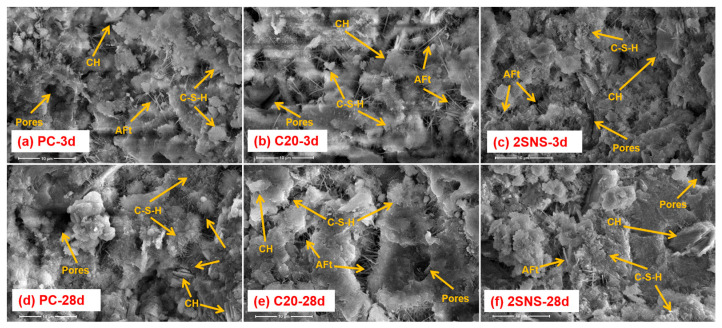
Microscopic image of the selected pastes after 1 and 28 d of curing.

The microscopic morphology of selected samples at different curing ages are shown in Figure 11. At 3 d, AFt, CH and C-S-H gels were produced in hydrated PC (Figure 11a). In addition, large quantities of pores were also noticeable, which were left by the free water. As shown in Figure 11b, the morphology of the hydrates changed significantly in C20, where some irregular metakaolin particles, more pores and a looser structure than that in PC can be observed. This also helps to explain the increased porosity (Figure 9) and decreased compressive strength (Figure 5) of C20 when CCG was added to cement at 3 d. Compared with the SEM image of C20, the microstructure of 2SNS (Figure 11c) was much denser due to the increased precipitation of hydrates caused by the addition of 2% SNS as discussed above, which also accounted for its much higher compressive strength at 3 d (Figure 5).

As hydration progressed up to 28 d (Figure 11d–f), the structural compactness of each sample increased significantly, as reflected by the reduction in the number of pores and overall porosity. This is because continuous reactions in these samples would produce more hydrates, such as carboaluminates and C-A-S-H gels. These hydrates would become closer, interact with each other and form a dense microstructure. Compared with PC (Figure 11d), a more compact morphology can be observed in 2SNS (Figure 11f), where the hydrates were closely linked together. However, the microstructure of C20 (Figure 11e) was still less compact than that of PC. These findings are well in line with the XRD, TG, MIP and compressive strength results above.

## 4. Conclusions

This paper investigated the effect of SNS particles on the workability, compressive strength, hydration performance and microstructural development of CCG-blended cements. Based on the experimental results above, the following conclusions can be drawn:

(1) The incorporation of SNS resulted in a significant reduction in the fluidity of CCG-blended mortar. This is due to the increased specific surface area of SNS, which needs more water to form lubricating layers around each particle and consequently decreased the fluidity of the mortar.
(2) SNS significantly improved the compressive strength of the CCG-blended cement at all curing ages, even compensating for the strength loss caused by the 20 wt.% replacement of PC by CCG. Furthermore, the optimum SNS content was found to be 2%, beyond which the compressive strength of CCG-blended cement would decrease.
(3) The addition of SNS promoted not only cement hydration at the early stage but also the reaction between CCG and CH at the late stage, which produced more hydrates than that in the CCG-blended paste.
(4) The addition of SNS could refine the pore structure of the CCG-blended paste, as evidenced by the decreased porosity and large capillary pores and the increased medium capillary pores. This refinement also contributed to the formation of a dense microstructure of the CCG-blended paste, offering advantages in the development of the compressive strength of the CCG-blended cement at all curing ages.

The findings of this study have important implications, as they suggest a new strategy to efficiently improve the early compressive strength of PC with CCG. However, further research and investigation into durability, including shrinkage, carbonation and chloride ion or sulphate attack, is also required to fully understand the benefits of SNS on the performance of PC with CCG.

## Figures and Tables

**Table 1:** Detailed mixture proportions for the mortar samples (g, using 100 g cement as the basis).

Code	Cement	CCG	SNS	Sand	Water
PC	100	0	0	300	50
C20	80	20	0	300	50
1SNS	79	20	1	300	50
2SNS	79	20	2	300	50
3SNS	79	20	3	300	50

## Data Availability

The data used in this study can be required from the corresponding author. The data are not publicly available due to information that could compromise the research participants’ privacy.
